# Inpatient Rehabilitation After Acute Severe Stroke: Predictive Value of the National Institutes of Health Stroke Scale Among Other Potential Predictors for Discharge Destination

**DOI:** 10.1177/27536351231157966

**Published:** 2023-05-20

**Authors:** Sinikka Tarvonen-Schröder, Tuuli Niemi, Mari Koivisto

**Affiliations:** 1 Neurocenter, Turku University Hospital, Turku, Finland; 2 Department of Clinical Neurosciences, University of Turku, Turku, Finland; 3 Department of Expert Services, Turku University Hospital, Turku, Finland; 4 Department of Biostatistics, University of Turku, Turku, Finland

**Keywords:** Discharge destination, National Institutes of Health Stroke Scale, predictor, rehabilitation, stroke

## Abstract

**Background:**

Research focusing on predictors for discharge destination after rehabilitation of inpatients recovering from severe stroke is scarce. The predictive value of rehabilitation admission NIHSS score among other potential predictors available on admission to rehabilitation has not been studied.

**Aim:**

The aim of this retrospective interventional study was to determine the predictive accuracy of 24 hours and rehabilitation admission NIHSS scores among other potential socio-demographic, clinical and functional predictors for discharge destination routinely collected on admission to rehabilitation.

**Material and Methods:**

On a university hospital specialized inpatient rehabilitation ward 156 consecutive rehabilitants with 24 hours NIHSS score ⩾15 were recruited. On admission to rehabilitation, routinely collected variables potentially associated with discharge destination (community vs institution) were analyzed using logistic regression.

**Results:**

70 (44.9%) of rehabilitants were discharged to community, and 86 (55.1%) were discharged to institutional care. Those discharged home were younger and more often still working, had less often dysphagia/tube feeding or DNR decision in the acute phase, shorter time from stroke onset to rehabilitation admission, less severe impairment (NIHSS score, paresis, neglect) and disability (FIM score, ambulatory ability) on admission, and faster and more significant functional improvement during the in-stay than those institutionalized.

**Conclusion:**

The most influential independent predictors for community discharge on admission to rehabilitation were lower admission NIHSS score, ambulatory ability and younger age, NIHSS being the most powerful. The odds of being discharged to community decreased with 16.1% for every 1 point increase in NIHSS. The 3-factor model explained 65.7% of community discharge and 81.9% of institutional discharge, the overall predictive accuracy being 74.7%. The corresponding figures for admission NIHSS alone were 58.6%, 70.9% and 65.4%.

## Introduction

Globally, stroke is the leading cause of adult disability.^1^ In Europe alone, it affects an estimated 1.1 million inhabitants every year and causes nearly half a million deaths.^2^ Around 40% to 50% of stroke survivors need multidisciplinary rehabilitation. Returning home is one of the main targets of stroke patients when admitted to a rehabilitation unit.^3^ Early community discharge is recommended.^4^ As discharge planning of patients with severe stroke is usually most challenging, early detection of discharge disposition and timely preparation of discharge procedures would allow a smooth patient- and family-centered transition and optimal use of resources.

Discharge disposition can be considered a key indicator of rehabilitation success. Factors predicting community discharge after inpatient rehabilitation include socio-demographic factors as well as clinical and functional characteristics like financial and environmental aspects, lesion location and type, impairment of ambulation or balance, cognitive impairment, neglect, sphincter incontinence, dysphagia, progress in activities of daily living and comorbidities.^5-12^ There is, however, no reliable prediction model to determine discharge disposition using the parameters available on admission. In numerous studies the most important and consistent predictor has been stroke severity or degree of disability assessed with various measures,^7,11,13^ most often with Functional Independence Measure (FIM).^7,10,14^ When exploring the consequences of stroke, it would be preferable, however, to determine severity with a stroke-specific measure like the National Institutes of Health Stroke Scale (NIHSS).^15^ In addition, NIHSS can be performed rapidly on admission in contrast to generic functional measures like FIM. Former studies have usually investigated predictors for discharge disposition among stroke patients or rehabilitants in general; acute NIHSS has been applied in some of these studies.^16,17^ However, patients with severe stoke admitted to subacute rehabilitation may have a different set and emphasis of predictors from the overall stroke population. Few studies focus specifically on severe stroke.^18-22^ Among patients with acute severe stroke, it has been shown that treating physicians’ estimation of functional outcome at 6 months is relatively inaccurate.^23^

Our hypothesis was that after acute severe stroke, reassessing neurological status applying NIHSS on admission to rehabilitation would be beneficial for estimating outcome. Thus, to identify predictors for discharge destination after inpatient rehabilitation, patients with severe neurological impairment in acute phase assessment were re-examined with the aim of determining the predictive value of rehabilitation admission NIHSS score in addition to acute NIHSS score and socio-demographic, clinical and functional factors easily detected and routinely collected in clinical practice.

## Patients and Methods

On a university hospital specialized inpatient rehabilitation ward, 156 consecutive stroke patients with severe stroke were enrolled between August 2015 and June 2021. The patients were mostly referred to the neurological rehabilitation unit from the acute stroke unit of the same university hospital. Sometimes the patient had to wait after the acute stroke unit care on a general ward for stabilization of the medical condition before intensive rehabilitation.

Inclusion criteria of this study were age over 18 years, premorbid independent living, major ischemic or hemorrhagic stroke demanding inpatient rehabilitation after acute care, 24 hours NIHSS ⩾ 15 denoting severe stroke,^24^ first time need for inpatient rehabilitation, and ability to sit in a wheelchair for a minimum of 30 minutes. Exclusion criteria were previous stroke causing permanent disability, current major medical, neurological or psychotic condition in addition to stroke, and medical reasons for interrupted rehabilitation.

The rehabilitation ward admitted post stroke patients cognitively and physically able to participate in intensive rehabilitation with the aim of possible community/home discharge. A multidisciplinary team evaluation included assessments made by a neurologist/rehabilitation physician, a physiotherapist, an occupational therapist, a neuropsychologist, a speech therapist, a social worker, a rehabilitation planner, rehabilitation nurses and when needed, also other consultants. Intensive comprehensive inpatient rehabilitation program consisted of combined coordinated meetings with these rehabilitation specialists 5 days a week according to patients’ individual needs in addition to constant daily rehabilitative nursing. The in-stay was restricted up to some weeks or a couple of months depending on the progress in rehabilitation. Because of lack of capacity of the rehabilitation ward, it was not possible to wait for transition to a suitable discharge destination on the ward. After discharge, further care and rehabilitation was arranged according to patients’ needs.

When necessary for community discharge, the social security services could offer home modifications (eg, for wheelchair users), official caregivers and other social and health care services for the resident to be able to live at home. Maximal caregiver services usually included 1 paid caregiver 8 hours a day or alternatively 4 caregiver visits (1-2 persons) a day and the rest of the time assistance when necessary (call service). Sometimes the rehabilitant had to wait a while for the home modifications to be finished either at a relative’s or friend’s (ie, significant other’s) home or at other interim residence organized by the social and health care professionals of the rehabilitant’s residential area. If inpatient care and rehabilitation were still recommended the rehabilitant was transferred to a local health care center or other care/rehabilitation facility organized by the local social and health care professionals. If community discharge was not considered possible because of the heavy burden of care, the local social and health care professionals were obliged to arrange promptly suitable long-term institutional care/residence for the inhabitant. Rehabilitants discharged either to a local health care center or other facility were inpatient care and rehabilitation was continued and those directly discharge to long-term institutional care were included in the institution group, other rehabilitants belonged to the community group.

Inpatient rehabilitation costs are modest/reasonable for the rehabilitants or can be free of charge due to lack of means of the rehabilitant. Social services offered home or in institution are either free or may have a reasonable cost, sometimes also depending on the income level of the resident. Nevertheless, all residents are entitled to get the basic services they need in spite of their assets or income level.

Caregivers, usually family members or relatives, sometimes a close friend (ie, significant others) were encouraged to participate in daily activities, in different therapy and social work sessions and in at least 1 meeting with the entire team to discuss the current medical and functional status of the rehabilitant and the future plans and goals including discharge destination. The rehabilitation program usually included at least 1 home-training visit during a weekend accompanied by caregiver(s), who filled in a questionnaire about the rehabilitants capabilities and needs during the home stay. Before the home-training weekend, the rehabilitation planner made a home visit accompanied with the rehabilitant, caregiver(s) and 1 or 2 other members of the multi-professional team. Finally, the rehabilitants and their significant others chose the discharge destination (community/home or institution) after discussions with the multi-professional team.

### Socio demographic and independent variables

Socio-demographic data (age, education, gender, cohabiting, and working status) were gathered from the participants, caregivers, and family members. The independent variables available on admission and possibly affecting discharge destination included: admission assessments of stroke severity, ambulatory ability, presence and severity of paresis, neglect and aphasia, presence of apraxia and depression. In addition, ICD-10 diagnosis (I63 brain infarction, I61 intracerebral hemorrhage) and date of diagnosis, type, and location of lesion, 24 hours NIHSS score after possible thrombolysis and/or thrombectomy, number of comorbidities, and Charlson index, acute phase dysphagia/tube feeding and do not resuscitate (DNR) decision were collected from the hospital medical records. Time from stroke onset to rehabilitation admission and length of stay in rehabilitation were calculated. The total number of comorbidities was counted, a procedure previously used to categorize comorbidities,^25^ and also the Charlson comorbidity index was calculated.^26^ Neurological status and NIHSS score were recorded for all patients on the day of admission to rehabilitation by a neurologist. The presence and severity of inattention was assessed when applying NIHSS. In addition, neglect was scored according to the Catherine Bergego Scale (CBS) by an occupational therapist on admission. The presence of depression and apraxia were based on clinical judgment.

### Functional variables

The functional variables included admission FIM total score and motor and cognitive sub-scores, dependence level (FIM stage), domain, and item scores. In addition, the impact of FIM efficiency and effectiveness during the rehabilitation in-stay were analyzed (Table 3). As part of the formal rehabilitation program, a rehabilitation nurse, trained and accredited in accordance with the Uniform Data System standards as a Functional Independence Measure (FIM^®^) rater, assessed the level of functional and cognitive abilities of each rehabilitant at admission and discharge with an electronic FIM tool (FIM^®^ version 5.2, Amherst, NY, USA).

### Outcome variables

The 2 discharge categories (community/home vs institution) were chosen as the dependent outcome variables.

### Scales

NIHSS (https://www.stroke.nih.gov>documents>NIH_Stroke_Scale_508.pdf) is a 15-item scale used to assess stroke severity and neurologic impairment on the levels of consciousness, language, neglect, visual-field loss, extraocular movement, motor strength, ataxia, dysarthria, and sensory loss. Ratings for each item are scored with 3 to 5 grades with 0 as normal. The total score varies from 0 to 42 (“normal functioning”—“coma”); total scores 1 to 4 can be regarded as mild, 5 to 14 as moderate, and ⩾15 as severe stroke. An experienced assessor can perform the assessment in even <8 minutes.^27^ NIHSS is sensitive and one of the most reliable and valid instruments of clinical measurement in stroke. As the population admitted to intensive subacute stroke rehabilitation does not include the most severe stroke survivors, the potential ceiling effect is not a concern.^16^

CBS is a measure of functional neglect in spontaneous behavior in personal, peri- and extra-personal space. It is based on direct observation of 10 real-life situations, that is, grooming, dressing, eating, mouth cleaning, gaze orientation, knowledge of limbs, auditory attention, moving (collisions), spatial orientation, and finding personal belongings. It captures mild neglect better than traditional paper-pencil tests. The total score 1 to 10 means mild, 11 to 20 moderate, and 21 to 30 severe neglect.^28,29^ Assessment time for a trained assessor is usually 30 or more minutes depending on the patient.

FIM is an 18-item assessment tool consisting of 2 main sub-scores (motor and cognitive) as well as 6 minor sub-scores: self-care (eating, grooming, bathing, dressing upper and lower body, and toileting), sphincter control (bladder and bowel management), transfers (bed/chair/wheelchair, toilet, and tub/shower), locomotion (walk/wheelchair/both and stairs), communication (comprehension and expression), and social cognition (social interaction, problem solving, and memory). Each item is measured on a 7-point scale ranging from 1 (total assistance) to 7 (complete independence). The total score ranges from 18 to 126, motor sub-score from 13 to 91, and cognitive sub-score from 5 to 35.^14^ For a trained professional, rating the items electronically takes about 60 minutes. The assessment is done within 3 days, the recording being performed usually on the second or third day after admission.

FIM efficiency is the average change in FIM score per day calculated as (FIM at discharge − FIM at admission) divided by the mean length of stay. FIM effectiveness is ([FIM at discharge − FIM at admission]/FIM at admission) × 100%. Corrected FIM effectiveness is calculated as FIM effectiveness/(A − FIM on admission); A is generally taken to be 126 points for total FIM score and 91 points for motor FIM score. This corrected version of FIM effectiveness corrects the ceiling effect present in FIM gain. FIM motor effectiveness with advanced correction corrects for both floor and ceiling effects and is calculated so that motor FIM effectiveness is around 0.65 whereupon A varies being 42, 64, 79, 83, 87, 89, or 91 points when the admission FIM motor sub-score is 13 to 18, 19 to 24, 25 to 30, 31 to 36, 37 to 42, 43 to 48, or 49 to 90 points, respectively (http://udsmr.org).

Patients have given their verbal and written informed consent for participation in the research study.

The Ethics Committee of the University and University Hospital approved the study (19.5.2015, 73/2015). The ethical standards of the World Medical Association Helsinki Declaration of 1975, as revised in 1983 were followed.

### Statistical analysis

Descriptive statistics were calculated for all variables and were presented as frequencies and percentages for categorical variables and as medians with range of values and IQR (interquartile range) for continuous variables. Between-group differences (community vs institution) for categorical variables were explored using Chi-squared test, or, in case of small frequencies, Fisher’s exact test, and for continuous variables using non-parametric Kruskal-Wallis test. Difference on Hodges-Lehmann estimate for median difference was used. The factors found to have a significant bivariate association (*P* < .05) with the outcome variable were included as independent variables in a logistic regression analysis. For a 1 unit increase in 1 independent variable at a time, the odds of getting discharged into the community were studied. Multivariable logistic regression analysis for community discharge was also conducted (Tables 5 and 6). Results were presented as odds ratios with 95% confidence limits. First multivariable model included 9 variables and second model included 8 variables that were chosen from variables that were statistically significant in univariate models. Bonferroni corrections were calculated. Since NIHSS score on admission, ambulatory ability, and age were found to be the most influential predictors in multivariable models, we conducted 1 model that included NIHSS score on admission, ambulatory ability and age, another model that included NIHSS score on admission and age, and finally a model with NIHSS on admission alone. The accuracy of 1-, 2-, and 3 factor models was reported as the overall percentage of correct classifications. Possible multicollinearity was checked; correlation coefficient ⩾.8 was considered a sign of multicollinearity. P-values below .05 (2-tailed) were considered statistically significant. SAS 9.4 for Windows (SAS Institute Inc., Cary, NC, USA) was used for all statistical analyses.

## Results

Of the 156 rehabilitants included in the study, 70 (44.9%) were discharged to community (home), and 86 (55.1%) were discharged to institutional care, which usually meant discharge to a residential health care center where assisted living residence or other long-term facility was arranged locally; sometimes, however, the permanent facility was arranged directly from the rehabilitation ward (n = 17).

Socio-demographic data of the 2 rehabilitant subgroups (community vs institution) are shown in Table 1. At stroke onset, 54 of the rehabilitants were working, 10 were unemployed and the rest 92 rehabilitants were retired, of them 34, 3, and 33 were discharged home, respectively. Significant differences between the 2 subgroups were found in age, working status, and years of education. Gender and living situation (cohabiting) were not significantly different between the subgroups.

**Table 1.** Socio-demographic data of the subgroups.

Variables	Community (n = 70)	Institution (n = 86)	Difference between medians (95% CL), *P*
Median (IQR, range)			
Age (y)	62.0 (53.0, 66.0; 16.0-80.0)	68.0 (59.0, 74.0; 38.0-83.0)	−6.5 (−10.0, −3.0), .0002
Education (y)	11.0 (9.0, 14.0; 6.0-20.0)	10.0 (8.0, 13.0; 6.0-22.0)	1 (0, 2), .03
N (%)			
Gender male	41 (58.6)	53 (61.6)	.74
Cohabiting	47 (67.1)	48 (55.8)	.19
Still working	34 (48.6)	20 (23.3)	.001

Abbreviations: CL, confidence limits; IQR, interquartile range.

Difference on Hodges-Lehmann estimate for median difference.

Significant between-group differences were found in several clinical and functional characteristics (Tables 2 and 3): 24 hours and admission NIHSS score, admission FIM total score and FIM grade, FIM motor and cognitive sub-score, all studied domain and item scores except for communication, FIM efficiency and corrected total and motor effectiveness and total effectiveness with advanced correction, time from stroke onset to rehabilitation, ambulatory ability (ambulation vs wheelchair use), severity (but not presence) of paresis and neglect (CBS), acute phase dysphagia and/or tube feeding, and DNR decision. Time since stroke onset to rehabilitation admission did not differ significantly between the 2 subgroups. Only 5 rehabilitants had longer than 6 months from stroke onset, 4 of them just over 6 months and one over 7 months, but under eight months. Two of these 5 were discharged to community.

**Table 2.** Clinical characteristics on admission (categorial variables) of the subgroups.

Variables N (%)	Community	Institution	*P*
	n = 70	n = 86	
Diagnosis			
Infarction	45 (64.3)	61 (70.9)	.38
ICH	25 (35.7)	25 (29.1)	
Localization			
Right hemispheral	21 (30.0)	25 (29.1)	.27
Left hemispheral	43 (61.4)	44 (51.1)	
Both sides	4 (5.7)	11 (12.8)	
Posterior circulation	2 (2.9)	6 (7.0)	
Ambulatory vs wheelchair	33 (47.1)/37 (52.9)	6 (7.0)/80 (93.0)	<.0001
Presence of paresis	69 (98.6)	85 (98.8)	1.0
Severity of paresis			
NIHSS 1, 2/3, 4/5, 6/7, 8/>8	3 (4.4)/7 (10.1)/18 (26.1)/39 (56.5)/2 (2.9)	0 (0.0)/3 (3.5)/13 (15.3)/60(70.6)/9 (10.6)	.01
Presence of sensory impairment	54 (77.1)	67 (77.9)	.9
Presence of neglect	59 (84.3)	79 (91.9)	.14
Severity of neglect			.04
No/mild/moderate/severe	10 (14.3)/44 (62.9)/9 (12.9)/7 (10.0)	7 (8.1)/41 (47.7)/23 (26.7)/15 (17.4)	
Presence of depression	5 (7.1)	15 (17.4)	.06
Presence of apraxia	36 (51.4)	48 (55.8)	.58
Presence of aphasia	50 (71.4)	57 (66.3)	.60
Severity of aphasia, NIHSS 1/2/3	9 (18.0)/20 (40.0)/21 (42.0)	7 (12.3)/23 (40.3)/27 (47.4)	.68
Dysphagia/tube feeding (acute)	38 (54.3)	64 (74.4)	.009
DNR (acute)	3 (4.3)	21 (24.4)	.0005
Number of comorbidities 0/1, 2/⩾3	13 (18.6)/47 (67.1)/10 (14.3)	10 (11.6)/58 (67.4)/18 (20.9)	.33
Charlson index 0/1, 2/⩾3	47 (67.1)/22 (31.4)/1 (1.4)	45 (52.3)/38 (44.2)/3 (3.5)	.17

Abbreviations: DNR, do not resuscitate; ICH, intracerebral hemorrhage; NIHSS, National Institutes of Health Stroke Scale; SAH, subarachnoid hemorrhage.

**Table 3.** Clinical characteristics and functioning (continuous variables) of the subgroups using Kruskal-Wallis test.

Variables median (IQR, range)	Community (n = 70)			Institution (n = 86)			Difference between medians (95% CL), *P*		
Time since stroke onset (d)	38	19, 66	6-219	63.5	35, 92	10-238	−20.0	−31, 9	.0005
Length of stay (d)	35.5	24, 48	4-102	31.5	18, 46	4-91	3	−3, 9	.35
NIHSS 24 h	17.5	15, 21	15-35	19	16, 22	15-38	−1	−2, 0	.03
NIHSS on admission	9	8, 12	1-19	12	10, 17	3-23	−3	−4, −2	<.0001
Severity of paresis on admission	4	2, 6	1-8	6	4.5, 8	1-14	−0.5	−1, 0	.004
CBS on admission	5	2, 10	0-30	9.5	4, 16	0-30	−2.75	−5, −0.5	.01
FIM on admission									
Dependence level	4	3, 5	2-6	3	2, 4	1-5	1.5	1, 2	<.0001
Total score	83.5	67, 97	38-119	57	43, 72	18-96	26.5	20, 33	<.0001
Motor subscore	59	41, 74	22-91	33.5	25, 46	13-74	21	15, 27	<.0001
Self-care (mean)	4.6	3.3, 5.5	1.8-7	2.8	2.2, 3.5	1-5.5	1.6	1.2, 2	<.0001
Sphincter control (mean)	5.5	4.5, 6.5	1-7	2.0	1, 5	1-7	2.5	1.5, 3.5	<.0001
Bladder	5.5	3, 7	1-7	1	1, 4	1-7	2.5	1, 4	<.0001
Bowel	6	5, 7	1-7	2.5	1, 6	1-7	2	1, 3	<.0001
Transfers (mean)	4	3, 6	1-7	2.3	1, 3.7	1-6	1.8	1, 2.7	<.0001
Locomotion	5	4, 6	1-7	4	2, 6	1-10	0.5	0, 1	.002
Stairs	1	1, 4	1-7	1	1, 1	1-5	0	0, 0	<.0001
Cognitive subscore	26	20, 29	6-34	20.5	14, 27	5-34	4.5	2, 7	.0006
Communication (mean)	5	3.5, 6	1-7	4	2.5, 6	1-7	0.5	0, 1	.09
Social cognition (mean)	5.7	4.3, 6	1.3-7	4.3	2.7, 5.3	1-6.7	1	0.7, 1.3	<.0001
FIM progress									
Efficiency	0.5	0.2, 0.7	−0.4-1.6	0.3	0.1, 0.5	−0.8-1.5	0.2	0.06, 0.3	.003
Effectiveness (%)	22.1	8, 42.0	−4.8-150.0	15.0	4.6, 38.0	−25.0-139.5	5.6	−1.5, 12.7	.13
Corrected total effectiveness	0.48	0.22, 0.65	−0.20, 0.88	0.15	0.04, 0.30	−0.39, 0.75	0.3	0.2, 0.4	<.0001
Corrected motor effectiveness	0.55	0.26, 0.70	−0.30, 1.0	0.18	0.04, 0.33	−1.12, 0.83	0.3	0.2, 0.4	<.0001
Motor effectiveness adv. corr.	0.58	0.26, 0.74	−0.30, 1.0	0.22	0.04, 0.40	−1.12, 1.14	0.3	0.2, 0.4	<.0001

Abbreviations: Adv corr, advanced correction; CBS, Catherine Bergego Scale; CL, confidence limits; FIM, Functional Independence Measure; IQR, interquartile range; NIHSS, National Institutes Health Stroke Scale.

Difference on Hodges-Lehmann estimate for median difference.

Table 4 demonstrates bivariate associations between community discharge and factors potentially affecting discharge destination. Significant associations were found in variables age, still working, time since stroke, 24 hours and admission NIHSS score, severity of paresis and neglect (CBS), all FIM admission scores except for communication, FIM efficiency and corrected total and motor effectiveness and total effectiveness with advanced correction, ambulatory ability, acute phase dysphagia, and/or tube feeding, and DNR decision. For every 1 year increase in age the odds of returning home decreased with 5.4%. For every 1 point increase in admission NIHSS score (range 0-42), the odds of returning home decreased with 16.1% and for every 1 point increase in total admission FIM score (range 13-126), the odds of returning home increased with 6.6%.

**Table 4.** Bivariate associations between community discharge and factors potentially affecting discharge destination.

Variables: OR (95% CL), *P*-value	Home vs institution		*P*
	OR	95% CL	
Continuous variables: median			
Age (1 y increase)	0.946	0.916, 0.976	.0005
Education (1 y increase)	1.063	0.971, 1.164	.19
Gender: male	0.880	0.462, 1.677	.70
Cohabiting	1.618	0.840, 3.116	.15
Still working	3.117	1.570, 6.186	.001
Time since stroke onset (1 d increase)	0.988	0.980, 0.996	.003
Length of stay (1 d increase)	1.005	0.988, 1.021	.57
NIHSS 24 h (1 point increase)	0.924	0.852, 1.001	.05
NIHSS on admission (1 point increase)	0.839	0.771, 0.913	<.0001
Severity of paresis (1 point increase)	0.795	0.671, 0.943	.008
CBS (1 point increase)	0.948	0.909, 0.988	.01
FIM on admission (1 point increase)			
Dependence level	2.892	2.022, 4.136	<.0001
Total	1.066	1.043, 1.088	<.0001
Motor	1.067	1.044, 1.091	<.0001
Self-care (mean)	2.750	1.930, 3.790	<.0001
Sphincter control	1.638	1.378, 1.947	<.0001
Bladder control	1.474	1.279, 1.699	<.0001
Bowel control	1.530	1.299, 1.802	<.0001
Transfers (mean)	1.821	1.471, 2.254	<.0001
Locomotion	1.318	1.098, 1.582	.003
Stairs	1.785	1.340, 2.377	<.0001
Cognitive	1.085	1.035, 1.136	.0006
Communication (mean)	1.190	0.992, 1.428	.06
Social cognition (mean)	1.653	1.298, 2.104	<.0001
FIM progress (increase)			
Efficiency	4.108	1.613, 10.466	.003
Effectiveness (%)	1.007	0.996, 1.017	.21
Corrected total effectiveness	55.139	12.551, 242.244	<.0001
Corrected motor effectiveness	42.548	10.651, 169.972	<.0001
Motor effectiveness with advanced correction	16.818	4.989, 56.686	<.0001
Categorical variables n (%)			
Diagnosis			
ICH vs infarction	1.356	0.690, 2.662	.38
Localization			.28
Right hemispheral vs posterior	2.520	0.459, 13.825	.24
Left hemispheral vs posterior	2.932	0.560, 15.336	.07
Both hemispheres vs posterior	1.091	0.153, 7.802	.38
Ambulatory vs wheelchair	11.889	4.584, 30.836	<.0001
Presence of paresis	0.811	0.050, 13.196	.88
Presence of sensory impairment	0.957	0.450, 2.037	.91
Presence of neglect	0.475	0.174, 1.299	.15
Presence of depression	0.364	0.125, 1.058	.063
Presence of apraxia	0.838	0.445, 1.579	.58
Presence of aphasia	1.272	0.641, 2.522	.49
Severity of aphasia			.69
Mild vs global	1.653	0.528, 5.171	.41
Moderate to severe vs global	1.118	0.489, 2.557	.74
Dysphagia/tube feeding (acute)	0.408	0.208, 0.802	.009
DNR (acute)	0.139	0.039, 0.487	.002
Number of comorbidities			.34
1-2 vs 0	0.623	0.251, 1.548	.89
⩾3 vs 0	0.4275	0.138, 1.323	.18
Charlson comorbidity index			.16
1-2 vs 0	0.554	0.285, 1.078	.98
⩾3 vs 0	0.319	0.032, 3.183	.47

Abbreviations: CBS, Catherine Bergego Scale; CL, confidence limits; DNR, do not resuscitate; FIM, Functional Independence Measure; NIHSS, National Institutes of Health Stroke Scale; OR, odds ratio.

Clinical assessment made on admission to rehabilitation except for acute NIHSS, dysphagia and DNR.

*P* < .05 is statistically significant.

Of the variables significant in bivariate analysis 9 factors in model 1 (Table 5) and 8 factors in model 2 (Table 6) were included in the multivariable regression analysis. Four explicative FIM items (grade, social cognition, bladder, and main locomotion, ie, ambulatory ability) were included in model 1, three (FIM total score, social cognition, and main locomotion) in model 2, the rest of FIM variables were excluded because of multicollinearity. FIM main locomotion was modified to ambulatory versus wheelchair-user by unifying the 6 participants using both ways to move with the sedentary subgroup. In clinical practice, this was also the reality on the day of admission. As FIM efficiency and corrected total and motor effectiveness had very large confidence limits they were excluded from the final analyses. In addition, these variables cannot be determined on admission to rehabilitation nor used for early prediction. Results of the final multivariable regression analysis are shown in Tables 5 and 6: lower admission NIHSS score, ambulatory ability and younger age were found to be the most influential predictors for discharge to community. This 3-factor model explained 65.7% of community discharge and 81.9% of institutional discharge. Overall predictive accuracy of this model was 74.7%. The figures for 2-factor model including NIHSS and age were 62.9%, 74.4%, and 69.2% and for NIHSS alone 58.6%, 70.9%, and 65.4%, respectively.

**Table 5.** Results of regression analysis with 9 variables significant in bivariate analysis: factors associated with community discharge.

Variables	OR	95% CL	*P*
Age	0.917	0.877, 0.958	.0001
Time since stroke	0.998	0.987, 1.008	.7
NIHSS on admission	0.863	0.759, 0.980	.02
Dysphagia (acute)	0.792	0.320, 1.965	.6
DNR (acute)	0.728	0.149, 3.564	.7
FIM grade (admission)	1.245	0.586, 2.645	.6
FIM social cognition (admission)	1.356	0.983, 1.960	.1
FIM bladder (admission)	1.119	0.872, 1.436	.4
Ambulatory (admission)	4.629	1.338, 16.018	.02

Abbreviations: CL, confidence limits; DNR, do not resuscitate; FIM, Functional Independence Measure; NIHSS, National Institutes of Health Stroke Scale; OR, odds ratio.

*P* < .05 is statistically significant.

**Table 6.** Results of regression analysis with 8 variables significant in bivariate analysis: factors associated with community discharge.

Variables	OR	95% CL	*P*
Age	0.917	0.878, 0.959	.0001
Time since stroke	0.998	0.988, 1.009	.8
NIHSS on admission	0.875	0.771, 0.994	.04
Dysphagia (acute)	0.799	0.322, 1.982	.6
DNR (acute)	0.660	0.137, 3.174	.6
FIM total (admission)	1.032	1.000, 1.066	.05
FIM social cognition (admission)	1.236	0.870, 1.757	.2
Ambulatory (admission)	3.811	1.109, 13.090	.03

Abbreviations: CL, confidence limits; DNR, do not resuscitate; FIM, Functional Independence Measure; NIHSS, National Institutes of Health Stroke Scale; OR, odds ratio.

*P* < .05 is statistically significant.

## Discussion

In this retrospective interventional cohort study of 156 consecutive inpatient rehabilitants recovering from acute severe stroke, 44.9% were discharged to community, and 55.1% were institutionalized. Those discharged to community were younger and more often still working, had less often dysphagia/tube feeding or a DNR decision in the acute phase, shorter time from stroke onset to rehabilitation admission, less severe impairment (NIHSS score, paresis, neglect) and disability (FIM score, ambulatory ability) on admission and faster and more significant functional improvement during the in-stay. The most significant independent predictors for community discharge in order of importance were lower admission NIHSS score, ambulatory ability on admission and younger age.

In the present study, the proportion of rehabilitants returning to community was in the range of previous studies on community discharge after acute severe stroke varying from 45%^20^ to 58%^18,30^ to 66%,^19^ in very severe stroke 36%.^18^ In accordance with our results, high age^7-9,17,19,31,32^ with rare exceptions,^33,34^ and high stroke severity and disability on admission to acute care (NIHSS) and on admission to subacute rehabilitation (FIM or Barthel Index)^11,13,14,17^ have been found to be the most important predictors of institutionalization after acute stroke care, after rehabilitation, and at 3 months after stroke. Likewise, older age^35^ and more severe stroke^35,36^ at acute discharge^36^ and in the first month and 3 or more months post-stroke^35^ have also been found to be significant predictors for overall poor stroke outcome, functional ability, and post-stroke disposition after rehabilitation.^36,37^ Furthermore, age, stroke severity, and disability have been found to be associated with each other.^36,38,39^ Like in the present study on admission to subacute rehabilitation, early walking ability after acute stroke care has been found to be a significant predictor of returning home, even independent of stroke severity.^40^

The present study showed that several other factors like time from stroke onset to rehabilitation, acute phase dysphagia and DNR decision, rehabilitation admission cognitive ability, paresis, and neglect severity, urinary, and bowel dysfunction, and functional improvement during rehabilitation were found to have bivariate association with discharge destination, but significance was not reached in multivariate analysis. Of these variables, cognitive impairment and dysphagia were found to be independent predictors for institutional discharge in a large American study in rehabilitants with varying stroke severities, other admission impairments like aphasia, ataxia, and neglect were found to be associated with discharge disposition only in bivariate analysis.^8^ Presence of dysphagia or tube feeding still at 3 months after stroke has also been found to be markers of poor functional and mortality outcomes.^41^ In line with our results, in previous studies on post stroke patients with mild to severe stroke,^7,9,10,35,42^ urinary and bowel incontinence have been found to be associated with discharge disposition, incontinence also with cognition and transfer FIM scores^44^ and severe post-stroke disability.^11^ In addition, early inpatient rehabilitation onset after acute care has previously been found to be associated with higher motor and cognitive improvement and community discharge^38,43^ like in the present study, but not always,^8^ and delayed admission, on the other hand, may be linked with longer length of stay,^43^ which has been associated with institutionalization.^37^ Also in line with our findings, higher motor and cognitive gain during rehabilitation has usually been found to have a positive effect on discharge disposition in patients with severe disability,^43-45^ but not always.^31^

The number of studies focusing specifically on post-rehabilitation discharge disposition in severe stroke is scarce compared to studies on discharge disposition after stroke in general, either after acute care or subacute rehabilitation. In studies on severe stroke, age,^19,30,46,47^ severity assessed with FIM,^19-21^ recovery assessed with Barthel Index,^46^ infections like pneumonia, urinary infection, and incontinence,^47^ length of rehabilitation in-stay, discharge ward, and geographical region^30^ and caregiver availability^18,19,46^ have been found to be associated with community discharge. Caregiver availability has also been found to be predictive of improved functional ability at discharge in individuals with severe stroke,^22^ thus promoting home discharge. However, it has been suggested, that family participation may promote home discharge and shorten length of stay in rehabilitation even in the absence of improving functioning per se.^48^ Nevertheless, cohabiting was not found to be associated with discharge disposition neither in the present study nor in a previous study in post stroke rehabilitants in the same facility.^9^ Even if there may be significant local differences in discharge practices due to socio-cultural and administrative factors and customs, there is strong evidence on similarities in predictors like age and severity level in different populations and nations.

Previous studies on discharge disposition after rehabilitation of in-patients with severe stroke have usually defined severity with FIM,^18-22^ sometimes with other measures like Barthel index,^23^ modified Rankin Scale,^47^ or length of in-stay.^30^ However, a stroke-specific measure like NIHSS allows assessing severity earlier, more precisely and rapidly on admission. NIHSS has been found to predict strongly recovery and outcome after acute stroke being superior to other stroke-specific measures.^16,49^ Previous studies on discharge disposition^13,15,16^ and associated factors^17,37^ have examined the utility of baseline NIHSS in acute settings^13,15,37^ or at 3 months from stroke onset.^16,17,49^ The present study is the first one to examine the utility of rehabilitation admission NIHSS score in addition to baseline (24 hours) NIHSS in predicting community discharge in rehabilitants recovering from acute severe stroke. In this population the 24 hours and rehabilitation admission NIHSS scores for those discharged to community and those institutionalized were on average 17.5 and 8, and 19 and 12, respectively. In a former study in the same facility consisting of over 200 consecutive post stroke rehabilitants with severity varying from mild to severe, the average baseline and rehabilitation admission NIHSS score in those discharged to community without assistance, with assistance and those institutionalized were 7 and 3, 11 and 7, and 17 and 11, respectively.^9^ Compared to these subacute rehabilitant cohorts, in a French population of more than 1000 consecutive acute stroke patients, those discharged to a nursing home, convalescent home, rehabilitation and home were found to have a clearly lower baseline NIHSS score of 12, 8, 7, and 3, respectively.^37^ In a meta-analysis, stroke survivors with baseline NIHSS score ⩾14 experienced discharge to institutionalized care 3.4 times more likely than home,^13^ the percentages being 50% to a nursing facility, 33% to rehabilitation, and 17% home,^15^ while in the present study 55% of subacute phase rehabilitants with baseline NIHSS ⩾ 15 were institutionalized after staying on average 1 month in rehabilitation, the rest returned home.

This study shows that rehabilitation admission NIHSS score on average 1 to 2 months after severe stroke is the most powerful predictor of post rehabilitation discharge destination explaining 58.6% of community discharge and 70.9% of institutional discharge, the overall predictive accuracy being 65.4%. In line with our results applying rehabilitation admission NIHSS, risk of nursing home placement 3 months after stroke onset has been found to increase sharply with age and baseline NIHSS in a Taiwanese registry study of more than 21 000 stroke inpatients. The patients with baseline NIHSS score 6 to 10, 11 to 15, 16 to 20, and >20 were found to have an odds ratio of 3.0, 6.6, 10.6, and 15.9 to be discharged to nursing home (instead of home) compared to those with a score 0 to 5. All other variables studied except for hypertension, that is, sex, diabetes mellitus, heart diseases, stroke history, snoring, lack of family member as a caregivers, and stroke types (main artery atherosclerosis or hemorrhage) were also found to be independent, but more modest predictors for institutionalization and gender difference disappeared in multiple logistic regression model.^17^ We found that for every 1 point increase in NIHSS score on admission to rehabilitation the odds of returning home on average 2 to 3 months after stroke onset decreased with 16.1% and for every 1 year increase in age the odds of being discharged home decreased with 5.4%. Together NIHSS score and age explained 62.9% of community discharge and 74.4% of institutional discharge, the overall predictive accuracy being 69.2%. Thus, it seems that NIHSS and age are highly influential predictors of discharge disposition not only after stroke care in general at 3 months^17^ but also after subacute inpatient rehabilitation in rehabilitants recovering from severe stroke, the rehabilitation admission NIHSS score being superior to acute (24 hours) NIHSS in the latter setting. Rehabilitation admission NIHSS score was superior to admission FIM total score in predicting discharge destination, too.

A Korean multicenter study on moderate stroke patients defined as NIHSS score 6 to 13 (mean 9.1) at discharge after acute rehabilitation (mean length of rehabilitation in-stay 32.5 days, at day 7 mean NIHSS score 14.2) is closest to the current study population even if the Korean patients had, on average, milder stroke. Of the Korean rehabilitants 51% were discharged home, while cognitive FIM sub-score, functional ambulation categories, modified Charlson comorbidity index and marital status were found to be independent predictors for home discharge.^50^ In the current study, 45% were discharged to community and the most powerful independent predictors for community discharge were lower stroke severity (NIHSS score) and ambulatory ability on rehabilitation admission, and younger age, the overall predictive accuracy of these 3 factors being 74.7%. Together they explained 65.7% of community discharge and 81.9% of institutional discharge. Cognitive FIM had a bivariate association with community discharge, but the significance disappeared in multivariate analysis. Comorbidities or co-habiting were not associated with discharge destination in the present study. As those discharged to community were younger, they were more often still working and possibly fewer were widowed, circumstances that can enhance returning home. However, socio-economic factors are not crucial in decision making as social insurance/security offers support for those who need services home including health care, home modifications, official caregiver services, and for example, transportation.

There are some potential limitations to this study. The number of rehabilitants was limited, but still adequate for the purpose of this retrospective research with no missing data. All eligible rehabilitants during a time period of almost 6 years were included. However, a more modest effect of some variables may not have reached significance. The data were obtained from a single academic hospital and therefore may not be generalizable to other settings. Nevertheless, research on a local scale has been recommended to compare possible differences in conducts and policies. It is noteworthy, that in Finland socio-economic factors do not play a critical role in discharge planning as community/state provides suitable housing and care when needed. Studies that examine discharge destination at a set interval after stroke have the advantage that the predictors are not affected by length of hospitalization. We, however, decided to investigate the discharge disposition at the time of hospital discharge (a real life situation) and to identify early predictors accordingly. The application of cross-sectional study design does not allow confirmation of causal relationships of disability. However, the inclusion of previously independent participants with first-ever stroke was based on a measure of stroke-related neurologic deficit (NIHSS), not on a functional tool like FIM, and comorbidities were assessed carefully. Other potential explanatory variables not used in the current study might also influence outcome; however, we did have a multitude of independent variables that are easily detected in clinical practice. Also, oversaturation of the regression model had to be avoided. Some variables, for example, contextual factors including living situation can be difficult to measure with adequate precision and some may be beyond the scope of this study.

## Conclusion

Subacute stroke rehabilitants with initially severe stroke discharged to community were younger and more often still working, had less often dysphagia/tube feeding or a DNR decision in the acute phase, had shorter time from stroke onset to rehabilitation admission, less severe impairment, and disability on admission to rehabilitation and faster and more significant functional improvement during the rehabilitation in-stay than those institutionalized. The most influential independent predictors for community discharge available on admission were lower rehabilitation admission NIHSS score, ambulatory ability on admission and younger age, NIHSS being the most powerful. NIHSS score alone explained 58.6% of community discharge and 70.9% of institutional discharge, the overall predictive accuracy being 65.4%. For every 1 point increase in NIHSS score the odds of being discharged to community decreased with 16.1%. The 3 factors together explained 65.7% of community discharge and 81.9% of institutional discharge, the overall predictive accuracy being 74.7%.

## References

[1] Feigin VL Stark BA Johnson CO , et al. Global, regional, and national burden of stroke and its risk factors, 1990-2019: a systematic analysis for the Global Burden of Disease Study 2019. Lancet Neurol. 2021;20:795-820. 34487721 10.1016/S1474-4422(21)00252-0 PMC8443449

[2] Wafa HA Wolfe CDA Emmett E Roth GA Johnson CO Wang Y. Burden of stroke in Europe. Stroke. 2020;51:2418-2427. 32646325 10.1161/STROKEAHA.120.029606 PMC7382540

[3] Gregory P Edwards L Faurot K Williams SW Felix AC. Patient preferences for stroke rehabilitation. Top Stroke Rehabil. 2010;17:394-400. 21131265 10.1310/tsr1705-394

[4] Winstein CJ Stein J Arena R , et al. Guidelines for adult stroke rehabilitation and recovery. Stroke. 2016;47:e98-e169. 10.1161/STR.0000000000000098 27145936

[5] Massucci M Perdon L Agosti M , et al. Prognostic factors of activity limitation and discharge destination after stroke rehabilitation. Am J Phys Med Rehabil. 2006;85:963-970. 17033592 10.1097/01.phm.0000242620.44924.1b

[6] Frank M Conzelmann M Engelter S. Prediction of discharge destination after neurological rehabilitation in stroke patients. Eur Neurol. 2010;63:227-233. 20215754 10.1159/000279491

[7] Saab A Glass-Kaastra S Young GB. Discharge destination from a rehabilitation unit after acute ischemic stroke. Can J Neurol Sci. 2019;46:209-215. 30739610 10.1017/cjn.2018.386

[8] Nguyen VQ PrvuBettger J Guerrier T , et al. Factors associated with discharge to home versus discharge to institutional care after inpatient stroke rehabilitation. Arch Phys Med Rehabil. 2015;96:1297-1303. 25823940 10.1016/j.apmr.2015.03.007

[9] Tarvonen-Schröder S Niemi T Koivisto M. Factors predicting discharge disposition after sub-acute stroke rehabilitation. Res Rep Med. 2020;3:1-12.

[10] Ouellette DS Timple C Kaplan SE Rosenberg SS Rosario ER. Predicting discharge destination with admission outcome scores in stroke patients. NeuroRehabilitation. 2015;37:173-179. 26484509 10.3233/NRE-151250

[11] Burton JK Ferguson EEC Barugh AJ , et al. Predicting discharge to institutional long-term care after stroke: a systematic review and metaanalysis. J Am Geriatr Soc. 2018;66:161-169. 28991368 10.1111/jgs.15101 PMC5813141

[12] Mees M Klein J Yperzeele L Vanacker P Cras P. Predicting discharge destination after stroke: a systematic review. Clin Neurol Neurosurg. 2016;142:15-21. 26802615 10.1016/j.clineuro.2016.01.004

[13] Thorpe ER Garrett KB Smith AM Reneker JC Phillips RS. Outcome measure scores predict discharge destination in patients with acute and subacute stroke: a systematic review and series of meta-analyses. J Neurol Phys Ther. 2018;42:2-11. 29232307 10.1097/NPT.0000000000000211

[14] Chumney D Nollinger K Shesko K Skop K Spencer M Newton RA. Ability of functional independence measure to accurately predict functional outcome of stroke-specific population: systematic review. J Rehabil Res Dev. 2010;47:17-29. 20437324 10.1682/jrrd.2009.08.0140

[15] Schlegel D Kolb SJ Luciano JM , et al. Utility of the NIH stroke scale as a predictor of hospital disposition. Stroke. 2003;34:134-137. 12511764 10.1161/01.str.0000048217.44714.02

[16] Muir KW Weir CJ Murray GD Povey C Lees KR. Comparison of neurological scales and scoring systems for acute stroke prognosis. Stroke. 1996;27:1817-1820. 8841337 10.1161/01.str.27.10.1817

[17] Tseng H-P Lin F-J Chen P-T , et al. Derivation and validation of a discharge disposition predicting model after acute stroke. J Stroke Cerebrovasc Dis. 2015;24:1179-1186. 25847306 10.1016/j.jstrokecerebrovasdis.2015.01.010

[18] Nguyen TA Page A Aggarwal A Henke P. Social determinants of discharge destination for patients after stroke with low admission FIM instrument scores. Arch Phys Med Rehabil. 2007;88:740-744. 17532895 10.1016/j.apmr.2007.03.011

[19] Pereira S Foley N Salter K , et al. Discharge destination of individuals with severe stroke undergoing rehabilitation: a predictive model. Disabil Rehabil. 2014;36:727-731. 24654961 10.3109/09638288.2014.902510

[20] Sandstrom R Mokler PJ Hoppe KM. Discharge destination and motor function outcome in severe stroke as measured by the functional independence measure/function-related group classification system. Arch Phys Med Rehabil. 1998;79:762-765. 9685088 10.1016/s0003-9993(98)90353-7

[21] Mokler PJ Sandstrom R Griffin M Farris L Jones C. Predicting discharge destination for patients with severe motor stroke: important functional tasks. Neurorehabil Neural Repair. 2000;14:181-185. 11272474 10.1177/154596830001400303

[22] Mirkowski M Pereira S Janzen S , et al. Caregiver availability for severe stroke results in improved functional ability at discharge from inpatient rehabilitation. Disabil Rehabil. 2018;40:457-461. 28006999 10.1080/09638288.2016.1260652

[23] Geurts M de Kort FAS de Kort PLM van Tuijl JH Kappelle LJ van der Worp HB. Predictive accuracy of physicians’ estimates of outcome after severe stroke. PLoS One. 2017;12:e0184894. 10.1371/journal.pone.0184894 PMC5621670 28961255

[24] Reznik ME Yaghi S Jayaraman MV , et al. Baseline NIH stroke scale is an inferior predictor of functional outcome in the era of acute stroke intervention. Int J Stroke. 2018;13:806-810. 29956598 10.1177/1747493018783759

[25] Silva AG Queirós A Sa-Couto P Rocha NP. Self-reported disability: association with lower extremity performance and other determinants in older adults attending primary care. Phys Ther. 2015;95:1628-1637. 26023215 10.2522/ptj.20140323

[26] Goldstein LB Samsa GP Matchar DB Horner RD. Charlson index comorbidity adjustment for ischemic stroke outcome studies. Stroke. 2004;35:1941-1945. 15232123 10.1161/01.STR.0000135225.80898.1c

[27] Brott T Adams HP Olinger CP , et al. Measurements of acute cerebral infarction: a clinical examination scale. Stroke. 1989;20:864-870. 2749846 10.1161/01.str.20.7.864

[28] Azouvi P Olivier S de Montety G Samuel C Louis-Dreyfus A Tesio L. Behavioral assessment of unilateral neglect: study of the psychometric properties of the Catherine Bergego scale. Arch Phys Med Rehabil. 2003;84:51-57. 12589620 10.1053/apmr.2003.50062

[29] Azouvi P Bartolomeo P Beis JM Perennou D Pradat-Diehl P Rousseaux M. A battery of tests for the quantitative assessment of unilateral neglect. Restor Neurol Neurosci. 2006;24:273-285. 17119304

[30] Teoh WL Finch E. Exploring discharge destination following severe stroke. Brain Impair. 2021;22:67-78.

[31] Koyama T Sako Y Konta M Domen K. Poststroke discharge destination: functional independence and sociodemographic factors in urban Japan. J Stroke Cerebrovasc Dis. 2011;20:202-207. 20621511 10.1016/j.jstrokecerebrovasdis.2009.11.020

[32] Saji N Kimura K Ohsaka G , et al. Functional independence measure scores predict level of long-term care required by patients after stroke: a multicenter retrospective cohort study. Disabil Rehabil. 2015;37:331-337. 24833418 10.3109/09638288.2014.918195

[33] Mutai H Furukawa T Araki K Misawa K Hanihara T. Factors associated with functional recovery and home discharge in stroke patients admitted to a convalescent rehabilitation ward. Geriatr Gerontol Int. 2012;12:215-222. 21929733 10.1111/j.1447-0594.2011.00747.x

[34] Hu HY Chi WC Chang KH , et al. The World Health Organization Disability Assessment Schedule 2.0 can predict the institutionalization of patients with stroke. Eur J Phys Rehabil Med. 2017;53:856-862. 28627860 10.23736/S1973-9087.17.04615-9

[35] van Almenkerk S Smalbrugge M Depla MF Eefsting JA Hertogh CM. What predicts a poor outcome in older stroke survivors? A systematic review of the literature. Disabil Rehabil. 2013;35:1774-1782. 23350761 10.3109/09638288.2012.756941

[36] Meyer MJ Pereira S McClure A , et al. A systematic review of studies reporting multivariable models to predict functional outcomes after post-stroke inpatient rehabilitation. Disabil Rehabil. 2015;37:1316-1323. 25250807 10.3109/09638288.2014.963706

[37] Béjot Y Troisgros O Gremeaux V , et al. Poststroke disposition and associated factors in a population-based study: the Dijon Stroke Registry. Stroke. 2012;43:2071-2077. 22627984 10.1161/STROKEAHA.112.658724

[38] Tarvonen-Schröder S Matomäki J Laimi K. Factors associated with outcomes of inpatient stroke rehabilitation. Int J Ther Rehabil. 2018;25:34-40.

[39] Sohrabji F Bake S Lewis DK. Age-related changes in brain support cells: implications for stroke severity. Neurochem Int. 2013;63:291-301. 23811611 10.1016/j.neuint.2013.06.013 PMC3955169

[40] Louie DR Simpson LA Mortenson WB Field TS Yao J Eng JJ. Prevalence of walking limitation after acute stroke and its impact on discharge to Home. Phys Ther. 2022;102:eng00226230031-eng00290231538. 10.1093/ptj/pzab246 PMC8787995 34718796

[41] Souza JT Ribeiro PW de Paiva SAR , et al. Dysphagia and tube feeding after stroke are associated with poorer functional and mortality outcomes. Clin Nutr. 2020;39:2786-2792. 31866129 10.1016/j.clnu.2019.11.042

[42] Kushner DS Johnson-Greene D. Association of urinary incontinence with cognition, transfers and discharge destination in acute stroke inpatient rehabilitation. J Stroke Cerebrovasc Dis. 2018;27:2677-2682. 29941393 10.1016/j.jstrokecerebrovasdis.2018.05.028

[43] Wang H Camicia M DiVita M Mix J Niewczyk P. Early inpatient rehabilitation admission and stroke patient outcomes. Am J Phys Med Rehabil. 2015;94:85-100. quiz 7-100. 25569470 10.1097/PHM.0000000000000226

[44] Bottemiller KL Bieber PL Basford JR Harris M. FIM scores, FIM efficiency, and discharge disposition following inpatient stroke rehabilitation. Rehabil Nurs. 2006;31:22-25. 16422041 10.1002/j.2048-7940.2006.tb00006.x

[45] Black TM Soltis T Bartlett C. Using the functional independence measure instrument to predict stroke rehabilitation outcomes. Rehabil Nurs. 1999;24:109-114. 21. 10.1002/j.2048-7940.1999.tb02151.x 10754896

[46] Jørgensen HS Reith J Nakayama H Kammersgaard LP Raaschou HO Olsen TS. What determines good recovery in patients with the most severe strokes? The Copenhagen Stroke Study. Stroke. 1999;30:2008-2012. 10512899 10.1161/01.str.30.10.2008

[47] Dutta D Thornton D Bowen E. Using population-based routinely collected data from the Sentinel Stroke National Audit Programme to investigate factors associated with discharge to care home after rehabilitation. Clin Rehabil. 2018;32:1108-1118. 29268625 10.1177/0269215517748715

[48] Hirano Y Maeshima S Osawa A , et al. The effect of voluntary training with family participation on early home discharge in patients with severe stroke at a convalescent rehabilitation ward. Eur Neurol. 2012;68:221-228. 22964874 10.1159/000338478

[49] Adams HP Jr Davis PH Leira EC , et al. Baseline NIH Stroke Scale score strongly predicts outcome after stroke. A report of the trial of org 10172 in Acute Stroke Treatment (TOAST). Neurology. 1999;53:126-131. 10408548 10.1212/wnl.53.1.126

[50] Kim MS Joo MC Sohn MK , et al. Development and validation of a prediction model for home discharge in patients with moderate stroke: the Korean stroke cohort for functioning and rehabilitation study. Top Stroke Rehabil. 2020;27:453-461. 31941411 10.1080/10749357.2019.1711338

